# Narrow band imaging (NBI) during medical thoracoscopy: first impressions

**DOI:** 10.1186/1745-6673-4-24

**Published:** 2009-08-26

**Authors:** Nicolas Schönfeld, Carsten Schwarz, Jens Kollmeier, Torsten Blum, Torsten T Bauer, Sebastian Ott

**Affiliations:** 1 Lungenklinik Heckeshorn, HELIOS Klinikum Emil von Behring, Berlin, Germany

## Abstract

**Background:**

This is the first ever evaluation of narrow band imaging (NBI), an innovative endoscopic imaging procedure, for the visualisation of pleural processes.

**Methods:**

The pleural cavity was examined in 26 patients with pleural effusions using both white light and narrow band imaging during thoracoscopy under local anaesthesia.

**Results:**

In the great majority of the patients narrow band imaging depicted the blood vessels more clearly than white light, but failed to reveal any differences in number, shape or size. Only in a single case with pleura thickened by chronic inflammation and metastatic spread of lung cancer did narrow band imaging show vessels that were not detectable under white light.

**Conclusion:**

It is not yet possible to assess to what extent the evidence provided by NBI is superior to that achieved with white light. Further studies are required, particularly in the early stages of pleural processes.

## 

Thoracoscopy is the standard diagnostic procedure for investigating exudative pleural effusions and leads to a conclusive diagnosis for 95% of patients when carried out under local anaesthesia [1]. Thoracoscopy can also be employed for staging primary thoracic malignancies, i.e. malignant pleural mesotheliomas or primary malignant pulmonary tumours with possible pleural dissemination. Despite the high diagnostic yield of thoracoscopy under local anaesthesia, some patients still remain without a conclusive diagnosis or have to undergo a surgical procedure under general anaesthesia. Apart from the conventional white light, other imaging procedures that are said to yield more information, especially at to the presence of a pleural tumour, have already been investigated, but the evidence has remained limited [2-4].

Narrow band imaging (NBI) is a new, alternative light-wavelength capture system that takes advantage of altered blood vessel morphology. Wavelengths of light in the visible spectrum are filtered from the illumination source, with the exception of narrow bands in the blue and green spectrum centered at 415 nm and 540 nm, coinciding with the peak absorption spectrum of oxyhemoglobin, making blood vessels more pronounced when viewed in NBI mode [5]. We present the first ever results with NBI in a series of patients with pleural processes.

## Methods

Medical thoracoscopy was performed under local anaesthesia and conscious sedation, using a prototype OLYMPUS XLTF-160 pleuravideoscope in single hole technique [6]. Following removal of the pleural fluid, the pleural cavity was inspected at first under white light and then under NBI as described elsewhere for bronchoscopy [5,7]. Afterwards, biopsies were taken from macroscopically altered sites. We used the OLYMPUS EVIS EXERA II video system (CV-180 videoprocessor and CLV-180 light source) manufactured by Olympus Medical Systems Corp., Japan. The findings were analysed retrospectively.

## Results

**Figure 1:**
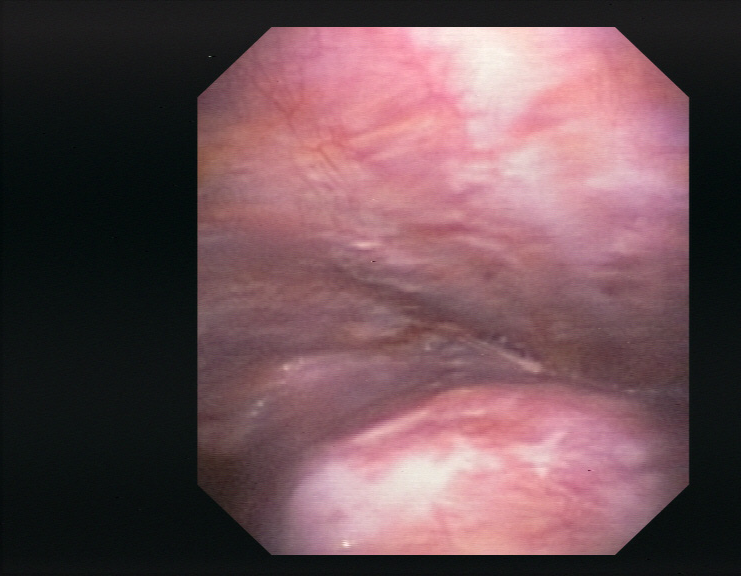
**Pleural cavity of patient #26 (lung cancer (adenocarcinoma), chronic inflammatory changes), white light**.

**Figure 2:**
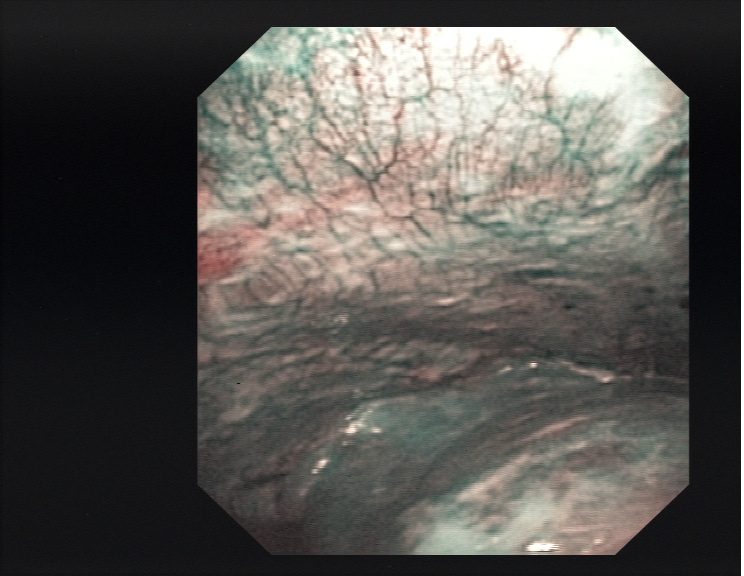
**Pleural cavity of patient #26, NBI**.

**Figure 3:**
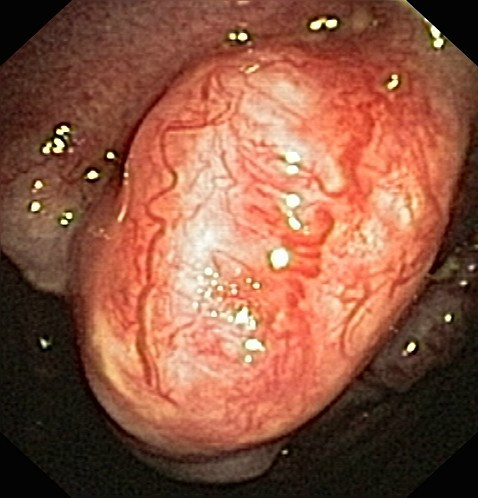
P**leural cavity of patient #2 (small cell lung cancer, large polyps), white light**.

**Figure 4:**
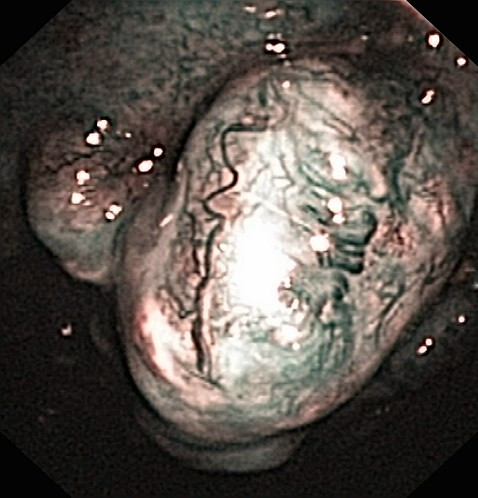
**Pleural cavity of patient #2, NBI**.

The results are summarised in Table 1. A total of 15 women (median age 66 years) and 11 men (median age 64 years) with pleural effusions were examined. Biopsies of the parietal pleura or diaphragm were taken for all but one of these patients. Only in patient #26 NBI showed more vessels than white light (fig. 1 and 2). In all other patients, there was either no difference, or blood vessels merely appeared more prominent (example in fig. 3 and 4).

**Table 1:** Results of thoracoscopy in all patients (n = 26)

Pat.	Gender	Age	Macroscopical findings	Histological diagnosis
1	female	81	chronic inflammation, pleural thickening (visceral and parietal)	chronic pleuritis (underlying disease: chronic renal failure)
2	male	74	multiple polyps (parietal)	small cell lung cancer
3	male	69	small nodes (parietal), adhesions	squamous-cell lung cancer
4	female	62	Adhesions, small nodes (parietal)	malignant mesothelioma
5	female	58	small confluent nodes (parietal)	breast cancer
6	male	59	pleural thickening (parietal)	lung cancer (adenocarcinoma)
7	female	81	large nodes (parietal and visceral), adhesions	malignant mesothelioma
8	male	47	large nodes (parietal), adhesions	malignant mesothelioma
9	female	65	small confluent nodes (parietal)	breast cancer
10	female	63	solitary node/polyp (parietal)	ovarian cancer
11	female	68	acute inflammation, adhesions, lymphangiectasis	breast cancer
12	male	64	small confluent nodes, polyps (parietal)	malignant mesothelioma
13	male	85	large nodes, polyps (parietal and visceral)	malignant mesothelioma
14	male	82	pleural plaques (parietal), adhesions	squamous-cell lung cancer
15	male	64	pleural plaques (parietal), small confluent nodes	malignant mesothelioma
16	female	88	multiple large nodes (parietal and visceral)	lung cancer (adenocarcinoma)
17	female	37	acute inflammation, adhesions, pleural thickening	tuberculous pleurisy
18	female	46	no abnormalities	inflammatory changes (underlying diease: squamous-cell lung cancer, effusion e vacuo)
19	female	63	subacute inflammation, pleural thickening	malignant mesothelioma
20	female	63	large nodes, polyps (parietal and visceral)	lung cancer (adenocarcinoma)
21	female	82	large confluent nodes, polyps (parietal and visceral)	malignant mesothelioma
22	male	64	multiple small nodes, polyps (parietal and visceral)	malignant mesothelioma
23	male	72	pleural thickening, solitary polyps	squamous-cell lung cancer
24	male	66	pleural thickening, adhesions	small cell lung cancer
25	female	80	large solitary nodes (parietal)	breast cancer
26	female	67	large solitary nodes (parietal) and chronic inflammatory changes	lung cancer (adenocarcinoma)

## Discussion

Our first examinations of the pleural cavity with NBI have indicated that in cases with diffuse spread of malignant tumour no substantial improvement in diagnoses is to be expected. Whereas the blood vessels in the region of the tumour tissue that was already identifiable macroscopically were more clearly depicted, the number of changes rendered visible was no greater than with white light. This was also true for mesothelioma patients. In the two cases of non-specific pleuritis, in which the pleura did not appear to be essentially thickened, the visualisation of the blood vessels was similar under both white light and NBI.

As was to be expected, NBI also failed to demonstrate any blood vessels in the deeper layers of pleural plaques typical of asbestos-related disease. However, a different situation was found in a single patient with pleura showing chronic inflammatory changes, besides tumour polyps. In this case distinctly more deeper blood vessels were identifiable than under white light. To what extent this observation is indicative of an actual diagnostic advantage cannot, however, be ascertained on the basis of this initial series.

Other groups having used different procedures such as fluorescence techniques reported to have found more exact indications of spreading of malignant pleural mesotheliomas [3,4]. However, the numbers of patients participating in these studies were so small that it has not as yet been possible to produce reliable evidence. It is possible that the same applies to NBI, in so far as in some cases mesotheliomas are associated with the development of a considerable amount of fibrotic tissue [8] and may thus not be identifiable histologically in biopsies taken under medical thoracoscopy. In such cases imaging of vascular structures in deeper layers of a thickened pleura could give some indication of from where the biopsy should best be taken. However, until considerably larger numbers of patients have been examined with NBI this remains speculation.

A second interesting question that could be investigated in future clinical studies with NBI in pleural processes is whether NBI were to be used intra-operatively to inspect the pleura before planned resection of lung cancer [9]. This would facilitate detection of any previously unobserved pleural dissemination at other locations. It is already common in surgery for small effusions associated with primary pulmonary malignomas to begin the operation under thoracoscopy and only to perform thoracotomy and continue with the resection if there are no signs of pleural dissemination. If possible, studies of this kind should not – as is so often done with innovative techniques – be carried out at only a single centre, but be performed as prospective, multicentre studies. It would thus be possible to arrive at a more objective assessment of such innovative techniques.

## Abbreviations

NBI: narrow band imaging.

## Competing interests of authors

The authors declare that they have no competing interests.

## Authors' contributions

All authors have taken part in the procedures (thoracoscopies) and, thus, the interpretation of clinical and endoscpical findings. Drs. Schönfeld, Bauer und Ott have in particular contributed to the retrospective analysis and interpretation of data.
